# Validation of suitable normalizers for miR expression patterns analysis covering tumour heterogeneity

**DOI:** 10.1038/srep39782

**Published:** 2017-01-04

**Authors:** C. Morata-Tarifa, M. Picon-Ruiz, C. Griñan-Lison, H. Boulaiz, M. Perán, M. A. Garcia, J. A. Marchal

**Affiliations:** 1 Biopathology and Medicine Regenerative Institute (IBIMER), University of Granada, Granada, Spain; 2 Biosanitary Institute of Granada (ibs.GRANADA), University Hospitals of Granada-University of Granada, Granada, Spain; 3 Braman Family Breast Cancer Institute, Sylvester Comprehensive Cancer Center, University of Miami, Miller School of Medicine, Miami, Florida, USA; 4 Department of Human Anatomy and Embryology, University of Granada, Granada, Spain; 5 Department of Health Sciences, University of Jaén, Jaén, Spain; 6 Virgen de las Nieves University Hospital, Oncology Department, Oncology Unit, Granada, Spain

## Abstract

Oncogenic microRNAs (miRs) have emerged as diagnostic biomarkers and novel molecular targets for anti-cancer drug therapies. Real-time quantitative PCR (qPCR) is one of the most powerful techniques for analyzing miRs; however, the use of unsuitable normalizers might bias the results. Tumour heterogeneity makes even more difficult the selection of an adequate endogenous normalizer control. Here, we have evaluated five potential referenced small RNAs (*U6, rRNA5s, SNORD44, SNORD24* and *hsa-miR-24c-3p*) using RedFinder algorisms to perform a stability expression analysis in i) normal colon cells, ii) colon and breast cancer cell lines and iii) cancer stem-like cell subpopulations. We identified *SNORD44* as a suitable housekeeping gene for qPCR analysis comparing normal and cancer cells. However, this small nucleolar RNA was not a useful normalizer for cancer stem-like cell subpopulations versus subpopulations without stemness properties. In addition, we show for the first time that *hsa-miR-24c-3p* is the most stable normalizer for comparing these two subpopulations. Also, we have identified by bioinformatic and qPCR analysis, different miR expression patterns in colon cancer versus non tumour cells using the previously selected suitable normalizers. Our results emphasize the importance of select suitable normalizers to ensure the robustness and reliability of qPCR data for analyzing miR expression.

MicroRNAs (miRs) are involved in the regulation of many physiological processes, including development, apoptosis and cell growth; as well as in pathological processes such as cancerogenesis^1^. Aberrant expression of miRs has been associated with tumour initiation, progression and patient outcome^2^,^3^. Since miRs are stable in tissues and blood plasma^4^, oncogenic miRs have emerged as diagnostic biomarkers and novel molecular targets for anti-cancer drug therapies^5^. Real-time quantitative PCR (qPCR) is one of the most powerful techniques for analyzing miR expression because of its sensitivity and specificity^6^,^7^,^8^. qPCR analysis to test miR expression is based in the use of endogenous controls for results normalization, reliability and reproducibility. However, there are several factors, such as RNA quality and purity, manipulability errors, differential stability and heterogeneity of the sample, among others, that may introduce variations in qPCR results. Moreover, for human samples it should be taken in account the endogenous variations of the biological individuals to avoid an erroneous interpretation of the data^9^. In addition, tumour heterogeneity makes even more difficult the selection of an adequate endogenous control. Housekeeping genes, ribosomal, small nuclear or nucleolar RNAs are frequently used as internal controls. However, according to experimental data, expression levels of these genes may differ in neoplastic and normal tissues^10^, and these variations may introduce bias to experimental results.

Tumour heterogeneity is characterized, in part, by different cell subpopulations including a small subset of cancer cells that act as initiating tumour cells or cancer stem cells (CSCs)^11^. This subpopulation maintains self-renewal, promotes cancer growth and it is responsible of drug/treatment resistance, tumour recurrence and metastasis^12^. Drug resistance and tumour recurrence in CSCs are mainly explained by the overexpression of multidrug resistance membrane proteins and the aldehyde dehydrogenase (ALDH) enzyme among others^13^. On the other hand, metastasis is one of the most crucial steps in cancer progression and the main cause of cancer-related mortality. Metastatic cancer cells are characterized for undergoing an epithelial-to-mesenchymal transition (EMT), losing their attachment to the epithelial niche and acquiring a mesenchymal phenotype^14^. ALDH activity has been extensively used for CSCs identification and isolation^15^. Recently, we have developed a non-aggressive, easy, inexpensive and reproducible methodology to isolate prospectively cancer stem-like cells based on their differential sensibility to trypsin exposure^16^. Trypsin-sensitive (TS) cancer cell subpopulation shows increased CSC properties when compared with the total population (TP) and/or the trypsin-resistant (TR) subpopulation^16^. Aberrant miR expression and the implication of these miRs on the biological complexities of CSCs, makes miR determination a powerful tool with great clinical potential, encouraging further studies with this approach. It has been well established that the use of a single or invalidated reference gene is not suitable to obtain reliable qPCR data^6^,^7^,^10^. The most well-known reference genes used in cancer are among others, *U6, rRNA5s, SNORD44* and *SNORD24*; however, their use has resulted in substantial discrepancies^10^ as we discuss in the next section. The aim of this study was to validate small RNAs as suitable normalizers comparing the expression of selected miRs between several human cancer cell lines, a normal (non-tumour) epithelial human cell line and enriched CSCs subpopulations isolated by ALDH activity and differential trypsinization methodologies. For this purpose, key points to obtaining reliable qPCR data were evaluated by Bestkeeper, NormFinder and comparative ΔCt stability methods. Moreover, we used miRanda-mirSVR, TargetScan 6.0 and Pictar bioinformatic analysis to predict miRs involved in self-renewal pathways and others cancer related pathways differently expressed in cancer cells versus non tumour cells.

## Results

### Determination of the best normalizer for miR expression in tumour cells and non-tumour cells

We evaluated the expression pattern of the most commonly used small RNAs normalizers in HT-29 and HCT-116 colon cancer cell lines and in the CCD-18Co normal colon established cell line. We selected *U6, rRNA5s, SNORD44* and *SNORD24*. We also used the *hsa-miR-24c-3p* since in some cases it has shown stability between several cell subpopulations^17^.

**Figure 1:**
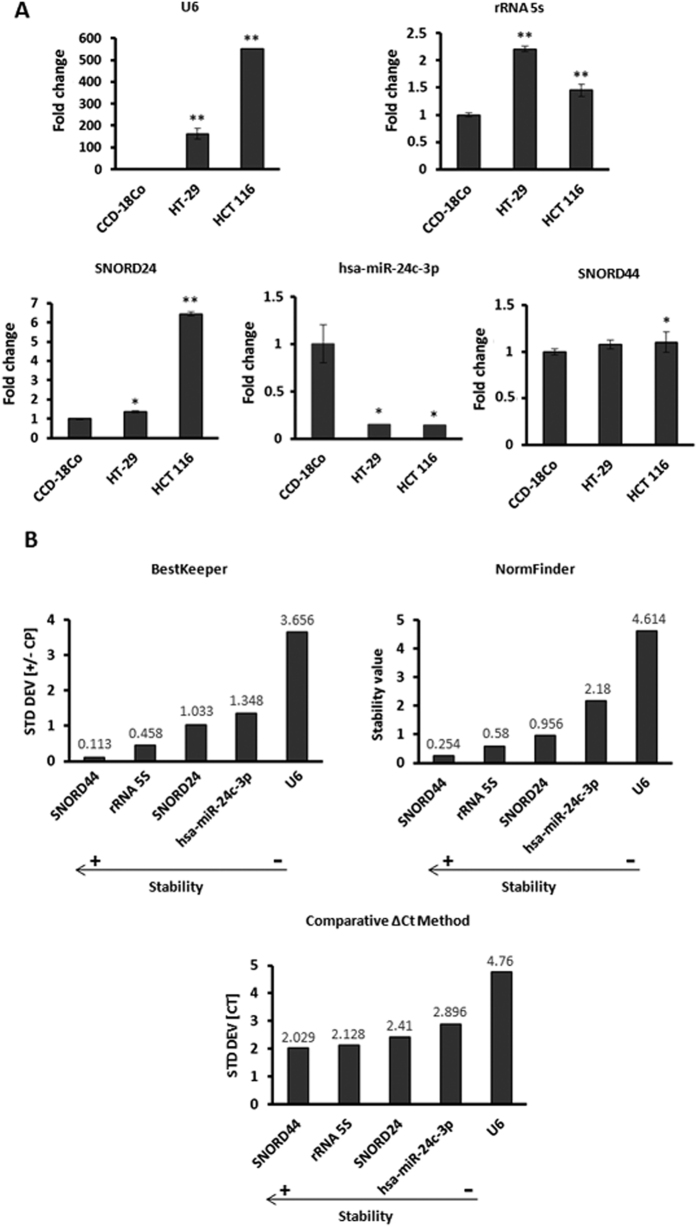
Relative expression and stability of five non-coding RNAs candidate reference genes for miR qPCR analysis in HCT-116 and HT-29 colon cancer cell lines versus CCD-18Co normal colon cell line. (**A**) Fold change of *U6, rRNA5s, SNORD24. hsa-miR-24c-3p and SNORD44.* Values were normalized using the UniSp6 RNA Spike-in control primer set. Data are mean values ± SEM of three independent experiments. Significance was calculated using Student’s *t*-test. *p < 0.05; **p < 0.01. (**B**) The stability of *U6, rRNA5s, SNORD24, hsa-miR-24c-3p and SNORD44* was determined by BestKeeper, Normfinder and comparative ΔCt method algorithms.

We compared miR expression levels relatively normalized to the artificial *UniSp6 RNA spike* from C. elegans. As shown in Fig. 1A, *U6* was significantly overexpressed up to 500-fold (p < 0.01) in HCT-116 and up to 100-fold (p < 0.01) for HT-29 colon cancer cell lines in comparison with normal colon cell line. The *rRNA5s* also displayed increased expression levels in both HT-29 and HCT-116 colon cancer cell lines up to 2.3-fold (p < 0.01) and 1.48-fold (p < 0.01), respectively. Moreover, *SNORD24* was significantly overexpressed in HT-29 (1.4-fold, p < 0.05) and HCT-116 (6.1-fold, p < 0.01) colon cancer cell lines in comparison with the CCD-18Co normal colon cell line. In contrast, the *hsa-miR-24c-5p* was significantly downregulated in both HT-29 and HCT-116 respect to the CCD-18Co normal colon cell line. Interestingly, *SNORD44* did not show significant differences in colon cancer cell lines in comparison with epithelial colon normal cells (Fig. 1A). Next, we evaluated the expression stability of the endogenous candidate normalizers using the values generated by Bestkeeper, NormFinder and comparative ΔCt methods and comparing normal cells versus cancer cells (Fig. 1B). Whereas *U6, rRNA5s, SNORD24* and *hsa-miR-24c-3p* were not suitable normalizers for colon cell lines, *SNORD44* showed high expression stability between tumour and normal colon cell lines (Fig. 1B).

### Determination of a suitable endogenous normalizer for cancer stem-like cell subpopulations

For the selection of small RNAs that could be used as normalizers in qPCR analysis of miR expression for cancer stem-like cell subpopulations, we analyzed two cell subpopulations with stemness properties: i) ALDH^+^ sorted cells by FACS and ii) low adherent cells isolated by differential trypsinization, as we have previously described in Morata-Tarifa *et al*. 2016^16^. We compared the expression of selected miRs between those two enriched CSC subpopulations, HCT-116 ALDH^+^ human colon cancer cells and MDA-MB-231 trypsin sensitive (TS) human breast cancer cells, with their corresponding ALDH^−^ and trypsin resistant (TR) subpopulations with not stemness properties. The artificial *UniSp6 RNA spike* from C. elegands was used as control. Since *SNORD24* showed a weak expression in these cell subpopulations, it was discarded as a potential housekeeping candidate.

**Figure 2:**
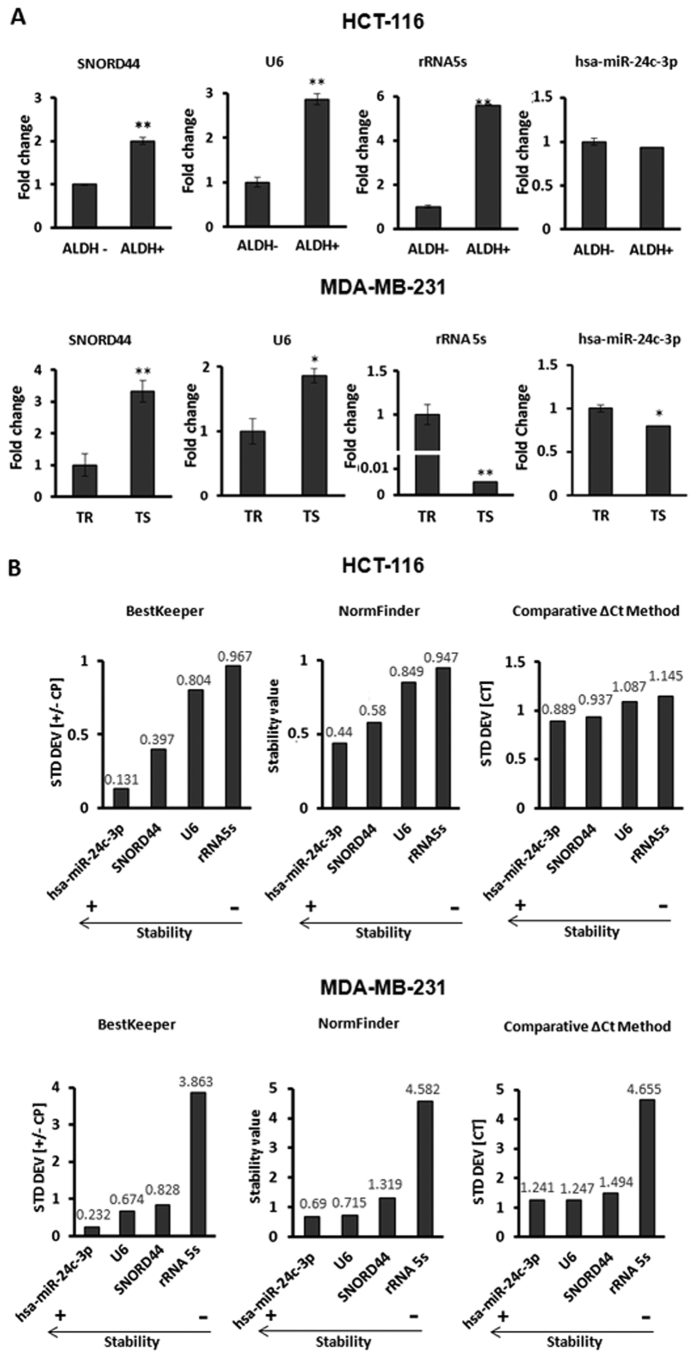
Relative gene expression and stability of the selected non-coding RNAs candidate reference genes for miR qPCR analysis in colon and breast cancer stem-like cell subpopulations *versus* the respective subpopulations without stemness properties. (**A**) Fold changes for *SNORD44, U6, rRNA5s* and *hsa-miR-24c-*3p in ALDH + versus ALDH- cells subpopulations from HCT-116 colon cancer cell line and in trypsin sensible (TS) versus trypsin resistant (TR) subpopulations from MDA-MB-231 breast cancer cell line. Values were normalized using the UniSp6 RNA Spike-in control primer set. Data are mean values ± SEM of three independent experiments. Significance was calculated using Student’s *t*-test. *p < 0.05; **p < 0.01. (**B**) Stability of *SNORD44, U6, rRNA5s* and *hsa-miR-24c-3p* was determined by both BestKeeper and Normfinder computational programs and comparative ΔCt method algorithms in HCT-116 and MDA-MB-231 subpopulations.

Firstly, *SNORD44* was analyzed since it was the one showing greater stability between tumour and non tumour cell lines (Fig. 1). However, *SNORD44* was significantly over-expressed in the ALDH^+^ subpopulation in comparison to ALDH^−^ subpopulation obtained from HCT-116 cell line (Fig. 2A). Similarly, *SNORD44* also showed a significantly higher expression in the TS subpopulation in comparison to the TR subpopulation selected from MDA-MB-231 cell line (Fig. 2B). Therefore, we decided to analyze other commonly used small RNA normalizers such as *U6*. However, this small RNA also showed a significantly higher expression in the ALDH^+^ and TS subpopulations regarding their respective non CSC ALDH^−^ and TR subpopulations (Fig. 2A). As in the two previous cases, *rRNA5s* significantly varied in the subpopulations analyzed showing higher expression in the ALDH^+^ subpopulation regarding the ALDH^−^ subpopulation in HCT-116 colon cancer cells (Fig. 2A). However, *rRNA5s* was downregulated in the TS breast cancer cell subpopulation in comparison to the TR subpopulation (Fig. 2A).

Finally, we found that *hsa-miR-24c-3p* did not show significant differences between ALDH^+^ CSC subpopulation when compared with ALDH^−^ non CSC subpopulation for colon cancer cells (Fig. 2A). Although a minimal statistical difference was found between TS CSC subpopulation and TR non CSC subpopulation from breast cancer cells (Fig. 2A), *hsa-miR-24c-3p* showed more stability than *SNORD44, U6* and *rRNA5s,* when were analyzed by Bestkeeper, NormFinder and comparative ΔCt methods, contrasting ALDH^+^ with ALDH^−^ subpopulations and TS with TR subpopulations, in both colon and breast cancer cells respectively (Fig. 2B).

### Expression patterns of miRs in several colon cell lines

**Figure 3:**
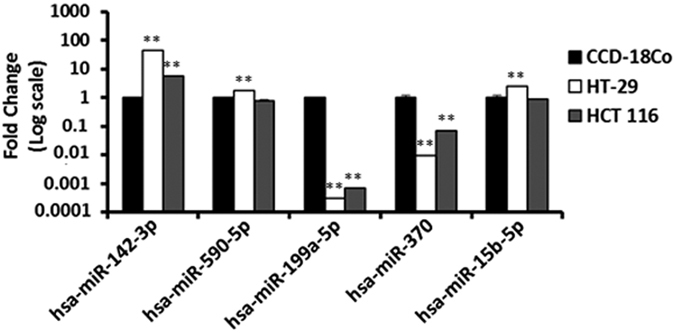
Relative gene expression of bioinformatic selected miRs differentially expressed by qPCR analysis in HCT-116 and HT-29 colon cancer cell lines *versus* CCD-18Co normal colon cell line. Values were normalized using the previously determined *SNORD44* normalizer. Data are mean values ± SEM of three independent experiments. Significance was calculated using Student’s *t*-test. *p < 0.05; **p < 0.01.

Once identified the most stable normalizer for miR expression analysis for each cell line or each CSC subpopulation, we decided to undertake a bioinformatic study for the identification of miRs targeting mRNAs involved in cancer pathways. We used miRanda-mirSVR (http://www.microrna.org/microrna/getGeneForm.do), TargetScan 6.0 (www.targetscan.org) and Pictar (http://pictar.mdc-berlin.de/) to identify predicted miRs targeting mRNAs involved in self-renewal pathways (Notch, Wnt and Hedgehog) and other CSC related pathways as shown in Supplementary Material and Methods section. We focused on 10 miRs identified by at least two of these applications and with better prediction algorithms (Table 1). The selected miR sequences are shown in Supplementary Table 1. These miRs were analyzed in colon cancer cell lines HCT-116 and HT-29 and in the non-tumor cells CCD-18Co (Fig. 3) using *SNORD44* as normalizer based on our previously described results. As shown in Fig. 3, tumour cell lines showed a significantly higher expression of *miR-142-3p*. However, *miR-590-5p* and *miR-15b-5p* were overexpressed in HT-29 cells, while in HCT-116 did not display significant changes. In addition, a dramatic decrease in *miR-199a-5p* and *miR-370* expression levels for the two tumour cell lines was observed when compared to the CCD18-Co non-tumour cell line. The analysis of selected miRs in cancer stem-like subpopulation using the *hsa-miR-24c-3p* normalizer was previously published in Morata-Tarifa C *et al*. 2016^16^.

## Discussion

Several studies based on miR expression profile have been performed in cell lines, normal and neoplastic tissues or in liquid biopsies to find diagnostic and therapeutic biomarkers for cancer^18^. Since small changes in the expression of a single miR may affect multiple genes, accurate measurement of miR expression is a critical prerequisite. However, some of the miR expression studies showed differing results suggesting that these discrepancies could be attributed to the different platforms and methods employed and the diversity of samples and control groups. Furthermore, the use of unsuitable reference genes seems to be one of the main reasons for the differences among the results obtained by qPCR studies. This fact highlights the importance of a correct standardization strategy in the qPCR analysis of miR profiles^10^,^19^. Currently, the majority of the studies investigating miR expression in cancer have used reference genes without a systematic validation of their stability in every model of cancer analysis. In fact, to the best of our knowledge, this is the first report detailing the identification and validation of suitable reference genes for miR qPCR assays, comparing colon cancer cells with the normal (non-tumour) colon cells. Moreover, we have also analyzed for the first time cancer stem-like cell subpopulations isolated by different methods in comparison with cancer cells without stemness properties. To do so, we performed a bioinformatic stability analysis to determine the most reliable normalizer for accurate miR profile studies in every experimental condition, evaluating a panel of five referenced small RNAs candidates such as *U6, rRNA5s, SNORD44, SNORD24* and *hsa-miR-24c-3p*.

Although *U6* and *rRNA5s* are commonly used in the normalization of miRs in different tumour samples^20^,^21^,^22^; however, in our study both miRs were upregulated in colon cancer cells respect to normal colon cells and in colon and breast cancer stem-like cells in comparison to cells without stemness properties. Therefore, these results suggest that both miRs could not be valid normalizers for studies comparing miR expression levels between non-malignant samples and cancer samples. For example, it has previously been shown that *U6* and *rRNA5s* were overexpressed in serum from breast cancer patients’ respect to healthy women’ serum^23^. Furthermore, *U6* expression levels in liver and breast cancer tissues were higher than those expressed in adjacent normal tissues^24^. These studies support the notion that the expression and distribution of *U6* and *rRNA5s* exhibits a high degree of variability among several types of human cells.

Although *SNORD24* has been recommended as an endogenous reference exhibiting the most stable expression levels between tissues and cells^25^ however, in our analysis *SNORD24* expression was significantly variable. In contrast, *SNORD44* expression level was similar in cancer and normal colon cell lines, although it was not suitable as normalizer for cancer stem-like subpopulations. In agreement with our results *SNORD44* has proved to be a stable normalizer in cancer and normal endometrial tissues^26^, although evidences that it is overexpressed in cancer respect to normal tissues have emerged^27^.

We also evaluated the expression stabilities of referenced genes using Bestkeeper, NormFinder and comparative ΔCt methods algorithms. These analyses showed that *SNORD44* was the most stable normalizer for studies with both cancer and normal colon cell lines. However, in qPCR analysis of cancer stem-like subpopulations, *SNORD44* was not the most stable normalizer. In fact *U6, rRNA5s* and *SNORD44* were overexpressed in the ALDH^+^ and TS subpopulations respect to the ALDH^−^ and TR subpopulations. Conversely, *hsa-miR-24c-3p* was the most stable miR among the several cancer stem cell subpopulations analyzed using both qPCR analysis and the three algorithms described. In fact, it has been previously reported that *hsa-miR-24c-3p* shows stability in CD44^+^ and CD44^−^ prostate cancer cell subpopulations^17^. In addition, it has been validated in bone marrow and peripheral blood samples from patients with neuroblastoma compared to healthy patients^28^, and used as normalizer for pancreatic ductal tumour assays^29^. Although *hsa-miR-24c-3p* was a valid normalizer for comparing cancer stem-like subpopulations, as we previously published^16^, however it did not show stability in tumour cells compared to normal cells. Therefore, our results encourage the need to set standards miR normalizers considering normal or cancer cell lines, different cell sub-populations or normal or cancer tissue samples.

Once identified the most stable normalizer we performed qPCR determinations of miRs identified through the bioinformatic analysis to validate its potential as candidate oncogenic miRs in cancer. Using *SNORD44* as normalizer in both HCT116 and HT-29 colon cancer cell lines, and in CCD-18Co normal colon cell line, we found that *miR-142-3p* was overexpressed in the colon tumour cells in comparison to normal cell line. *miR-142-3p* mediates Wnt signalling pathway activation in breast cancer through the inhibition of APC protein^30^, a protein that is also involved in intercellular adhesion and that is mutated in a high percentage of colorectal tumours^31^. Furthermore, *miR-590-5p* and *miR-15b-5p* were slightly but significantly elevated in the HT-29 cell line respect to non- tumour cells. Whereas it is widely known that *miR-15b* is elevated in samples from patients with colon cancer^32^,^33^, to our knowledge, this is the first implication of *miR-590-5p* overexpression in colon cancer cells. Moreover, it has been demonstrated that *miR-590-5p* is overexpressed in renal carcinoma cell lines^34^, hepatocellular^35^ and cervical cancer tissue^36^. On the other hand, we have identified a downregulation of *miR-199-5p* and *miR-370* in colon cancer cells, as it has been showed in other cancer types such as renal cancer, ovarian cancer, hepatocellular carcinoma, bladder cancer, and larynx cancer^37^,^38^,^39^,^40^. In addition, in endometrial ovarian cancer cells, *miR-370* suppressed cell viability and colony formation and increased chemosensitivity^41^, being also downregulated in chemoresistant breast cancer cells^42^. The analysis of selected miRs in cancer stem-like subpopulations using the *hsa-miR-24c-3p* normalizer was previously published in Morata-Tarifa C *et al*. 2016^16^.

In summary, our results encourage deeper analysis in miR patterns comparing cancer to healthy tissues, emphasizing the importance of a previous selection of suitable normalizers for each cancer, normal or cancer stem-like subpopulations or every tumour model of analysis. The selection of the best housekeeping will ensure the robustness and reliability of the results in precision or personalized medicine for cancer.

## Materials and Methods

### Cell lines and cell culture

Human colon cancer cell lines HCT-116 (ATCC CCL-247) and HT-29 (ATCC HTB-38), the triple negative human breast cancer cell line MDA-MB-231 (ATCC HTB-26), and the normal human colon cell line CCD-18Co (ATCC CRL1459) were obtained from American Type Culture Collection (ATCC) and cultured according to the ATCC indications.

### Differential trypsinization

Selection of cancer cells with CSC-like properties or with a more differentiated phenotype was performed using the differential trypsinization method as we previously described by Morata-Tarifa C *et al*. 2016^16^. Briefly, CSC-like cells were obtained based on their lower attachment capability to the cell culture surface by incubating cell cultures at 60–80% of confluence with 0.05% trypsin for 2 minutes at 37 °C. More differentiated cancer cells were isolated based on their resistance to detach from the cell culture surface by enzymatic digestion with diluted trypsin at 0.05%. These two different subpopulations, with differential phenotypic and functional properties, were named as Trypsin-Sensitive (TS) and Trypsin-Resistant (TR), respectively; its CSC properties were characterized as indicated by Morata-Tarifa *et al*. 2016^16^.

### Aldefluor assay

Aldehyde dehydrogenase (ALDH) enzyme activity in viable cells was assayed using Aldefluor^®^ kit assay (Stem Cell Technologies, Grenoble, France) according to the manufacturer’s instructions. Analyses were performed in a FACS CANTO II (BD Biosciences) cytometer using the FACS DIVA software.

### Bioinformatic miRs prediction

Predictions were realized looking for miRs targeting mRNA of proteins that enhance or inhibit CSC-related pathways (Supplementary information). miRs were selected based on three bioinformatics online tools: TargetScan 6.0., Pictar and miRanda-mirSVR. Predictions were selected based on the most favorable (lowest) context + score, higher probabilities and lowest sum of mirSVR scores, respectively^43^.

### RNA isolation and Quantitaitve Real Time Polimerase Chain Reaction (qPCR)

Total RNA, including miRs, was obtained from the different cell lines and subpopulations using the miRNeasy Mini Kit (Qiagen, Limburgo, Netherland) following manufacturer´s instructions. Reverse transcription from miRs was performed using the miRCURY LNATM Synthesis kit II (Exiqon, Vedbaek, Denmark) following manufacturer’s protocol. qPCR was performed using miRCURY LNA TM EXILENT SYBR Green (Exiqon) in a CFX96 real-time PCR detection system (Bio-Rad). Each reaction was performed in triplicate and the comparative threshold cycle (Ct) method was used to calculate the amplification factor. *UniSp6* RNA Spike-in control primer set is used to amplify *UniSp6* RNA Spike-in template for the control of the cDNA synthesis step. The standard curve was constructed by 5-fold serial dilutions of cDNA.

### Determination of stable normalizer RNAs

The stability of candidate reference miRs was evaluated using RefFinder (see Supplementary Material and Methods information), a user-friendly web-based comprehensive tool for evaluating and screening reference genes from extensive experimental datasets. RefFinder integrates the computational programs Normfinder, BestKeeper and comparative ΔCt method algorithms, to separately and comprehensively compare and rank the tested candidate reference genes with the data obtained for all subpopulations or cell types studied.

### Statistical analysis

All graphs present mean ± SEM from ≥ 3 assays. Student’s t-test was used for experiments with two groups. Comparisons of > 2 groups used one way analysis of variance (ANOVA) followed by Dunnett’s or Tukey’s post hoc analysis. Some experiments used two way analysis of variance followed by Tukey´s post hoc tests. P-values < 0.05 were considered statistically significant in all cases.

## Additional Information

**How to cite this article**: Morata-Tarifa, C. *et al*. Validation of suitable normalizers for miR expression patterns analysis covering tumour heterogeneity. *Sci. Rep.* **7**, 39782; doi: 10.1038/srep39782 (2017).

**Publisher's note:** Springer Nature remains neutral with regard to jurisdictional claims in published maps and institutional affiliations.

## Supplementary Material

Supplementary Information

## Figures and Tables

**Table 1:** List of miRs with the best prediction algorithm values.

Targets	miRs	TargetScan 6.0 (*Context* + *score*)	miRanda-mirSVR (*mirSVR score*)	PicTar (probability)
*NOTCH1*	*hsa-miR-34a*	−0.34	−1.2401	0.94
	*hsa-miR-34c*	−0.33	−1.2426	0.94
*NOTCH2*	*hsa-miR-15b*	−0.24		
	*hsa-miR-34a*	−0.13		0.87
	*hsa-miR-34c*	−0.12		0.89
*APH1B*	*hsa-miR-590*		−0.7131	
*MAML1*	*hsa-miR-93*			0.82
*RBPJ*	*hsa-miR-590*	−0.22	−0.9176	
	*hsa-miR-15b*		−0.9352	
*FZD4*	*hsa-miR-199a*	−0.34	−0.4930	0.85
*FZD5*	*hsa-miR-100*	−0.30		
*FZD6*	*hsa-miR-199a*	−0.43	−1.0331	0.97
*FZD8*	*hsa-miR-100*	−0.34	−1.1406	0.92
*FZD10*	*hsa-miR-15b*	−0.51	−1.3160	0.95
*AXIN*	*hsa-miR-15b*	−0.23	−0.8583	0.98
*GSK3B*	*hsa-miR-199a*	−0.20/−0.09		0.99
*APC*	*hsa-miR-142*	−0.40	−1.2461	
*PTCH1*	*hsa-miR-15b*	−0.24	−0.5850	
*SMO*	*hsa-miR-370*	−0.33/−0.26	−0.2491	
*CDKN1A*	*hsa-miR-93*		−0.6463	0.93
*ABCB5*	*hsa-miR-15b*		−1.2476	
*MYC*	*hsa-miR-34c*			0.99
