# Supplementary material for: Validation of suitable normalizers for miR expression patterns analysis covering tumour heterogeneity
Source: Sci Rep. 2017 Jan 4;7:39782. doi: 10.1038/srep39782 (PMC5209713; doi:10.1038/srep39782)
Supplement: Supplementary Information [file srep39782-s1.pdf]

Validation of suitable normalizers for miR expression patterns analysis covering tumour heterogeneity

Morata-Tarifa C[1,2,3], Picon-Ruiz M[1,2,3], Griñan-Lison C[1,2], Boulaiz H[1,2,4], Perán M[1,5], Garcia MA[1,2,6,*], Marchal JA[1,2,4,*]

[1]Biopathology and Medicine Regenerative Institute (IBIMER), University of Granada, Granada, Spain. [2]Biosanitary Institute of Granada (ibs.GRANADA), University Hospitals of Granada-University of Granada, Granada, Spain. [3]Braman Family Breast Cancer Institute, Sylvester Comprehensive Cancer Center, University of Miami, Miller School of Medicine, Miami, Florida, USA. [4]Department of Human Anatomy and Embryology, University of Granada, Granada, Spain. [5] Department of Health Sciences, University of Jaén, Jaén, Spain. [6]Virgen de las Nieves University Hospital, Oncology Department, Oncology Unit, Granada, Spain

*Corresponding authors: Juan Antonio Marchal Corrales jmarchal@ugr.es
María Angel Garcia Chaves mangelgarcia@ugr.es

Supplementary Information

Determination of stable housekeeping miRNAs using RefFinder

The stability of candidate reference miRNAs was evaluated using RefFinder integrating the computational programs Normfinder, BestKeeper and comparative ΔCt method algorithms. NormFinder combine intra- and intergroup variations, giving rise a stability value that represents a practical measure of systematic error that will be introduced when using the investigated gene (Andersen, Jensen et al. 2004). BestKeeper computes the descriptive statistics of the derived crossing points (CP) for each housekeeping gene (HKG) including the geometric mean (GM), arithmetic mean (AM), minimal (Min) and maximal (Max) value, standard deviation (SD), and coefficient of variance (CV). The x-fold over- or under-expression of individual samples towards the geometric mean CP are calculated and the multiple factor of their minimal and maximal values, expressed as the x-fold ratio and its standard deviation: Min[x_fold] = Emin[CP]−GM[CP]; Max[x_fold] = Emax[CP]−GM[CP]. The estimation of HKG expression stability is based on the inspection of calculated variations (SD and CV values). According to the variability observed, HKGs can be ordered from the most stably expressed, exhibiting the lowest variation, to the least stable one, exhibiting the highest variation. Any studied gene with the SD higher than 1 can be considered inconsistent (Pfaffl, Tichopad et al. 2004). On the other hand, the Comparative ΔCt method compares relative expression of 'pairs of genes' within each sample to identify useful housekeeping genes. If the ΔCt value between two genes remains constant, when is analyzed in different samples, it means either both genes are stably expressed among those samples, or co-regulated (here it is assumed the stability of both genes). However, if the ΔCt fluctuates, then 1 or both genes are variably expressed. Taking all the genes into account and by comparing all possible gene combinations, a pattern forms whereby genes tend to be associated with either increased or decreased levels of deviation in ΔCt among the samples, and hence, either an increase or decrease in the level of variability in gene expression.

Cancer-related pathways analyzed for bioinformatic miRNAs prediction

We first performer Notch route prediction looking for miRs targeting their specific receptors (NOTCH1-4) and other proteins involved in this pathway as *RBPJ*, DNER, MAML1, SNW1 (SKIP) and γ-secretase components, nicastrin (NCSTN), presenilin (PSEN1 and PSEN2), APH-1 (APH1A and APH1B) and PSENEN (PEN-2). The Wnt pathway predictions were done against their receptors (FZD 1-10), as well as CTNNB1 (catenin β-), WLS and negative regulators AXIN1 / 2, APC and GSK3B. For Hh route were performed the prediction of its receptors (PTCH 1/2), SMO, GLI1, GLI3 and BMI1. Other genes analyzed in the predictions were CDKN1A (P21), MYC and ABCB5. To make the selection we rely on the values obtained from context + score in TargetScan 6.0 and mirSVR score MIRANDA-mirSVR, being the most favorable those that have a lower value in analyzing cases. We also use the probability given by PicTar, being more favourable the greater value.

Supplementary table

Table 1S. Sequence of selected miRs

miRs	Sequence
hsa-miR-34a-5p	UGGCAGUGUCUUAGCUGGUUGU
hsa-miR-34c-5p	AGGCAGUGUAGUUAGCUGAUUGC
hsa-miR-15b-5p	UAGCAGCACAUCAUGGUUUACA
hsa-miR-590-5p	GAGCUUAUUCAUAAAAGUGCAG
hsa-miR-93-5p	CAAAGUGCUGUUCGUGCAGGUAG
hsa-miR-199a-5p	CCCAGUGUUCAGACUACCUGUUC
hsa-miR-100-5p	AACCCGUAGAUCCGAACUUGUG
hsa-miR-142-3p	UGUAGUGUUUCCUACUUUAUGGA
hsa-miR-370	GCCUGCUGGGGUGGAACCUGGU
